# Anaphylaxis to Kamut® flour in an adult patient: a case report

**DOI:** 10.1186/1710-1492-7-S2-A36

**Published:** 2011-11-14

**Authors:** Timothy J Olynych, Lori Connors

**Affiliations:** 1 Internal Medicine, Dalhousie University, Halifax, NS, Canada, B3J 3R4; 2 Clinical Immunology and Allergy, Dalhousie University, Halifax, NS, Canada, B3J 3R4

## 

Wheat products are nearly ubiquitous in society and are found in many food and non-food products. There are now a variety of Ancient grains that were previously not used in foodstuff in the Western diet. Kamutâ (khorasan wheat) flour is an ancient grain that was introduced in North America in the late nineties. It is commonly found in multigrain products.

Our patient is a 24-year-old female with a past medical history of well-controlled asthma. She describes oral itch and throat itch lasting 30 minutes with eating various types of multigrain products over several years. She eats whole wheat and white bread products without difficulty. After ingesting two pieces of pizza made with Kamutâ flour she developed chest tightness, back pain, vomiting, difficulty swallowing, and continued itch in her throat. Her symptoms resolved within an hour of taking an antihistamine. She underwent skin prick testing to the multiple bread products that she had reacted to in the past, as well as Kamutâ flour. Kamutâ flour had a 5mm and 7mm wheal with 22mm and 20mm flare respectively on two independent skin tests. Tests to the other multigrain products were also positive. After review of ingredient lists of the multigrain products she underwent further testing to a number of less commonly used flours and had positive tests to tritecare, quinoa and amaranth flours. She now carries an epinephrine injector and avoids multigrain and ancient grain products, including Kamutâ. To our knowledge this is the first case of anaphylaxis to Kamutâ flour.

